# Dietary and Genetic Aspects of Polycystic Ovary Syndrome (PCOS) in Polish Women—Part I: Nutritional Status and Dietary Intake

**DOI:** 10.3390/nu17142377

**Published:** 2025-07-21

**Authors:** Karolina Nowosad, Małgorzata Ostrowska, Paweł Glibowski, Katarzyna Iłowiecka, Wojciech Koch

**Affiliations:** 1 Department of Biotechnology, Microbiology and Human Nutrition, University of Life Sciences in Lublin, 8 Skromna Str., 20-704 Lublin, Poland; karolina.nowosad@up.lublin.pl (K.N.); malgorzata.ostrowska@up.lublin.pl (M.O.); 2 Nutrition Clinic, Department of Clinical Dietetics, Medical University of Lublin, 7 Chodzki St., 20-093 Lublin, Poland; katarzyna.ilowiecka@umlub.pl; 3 Department of Food and Nutrition, Medical University of Lublin, Chodźki 4a, 20-093 Lublin, Poland; wojciech.koch@umlub.pl

**Keywords:** PCOS, nutrition, body composition, phase angle, omega-3 fatty acids

## Abstract

**Background:** Polycystic ovary syndrome (PCOS) is a common endocrine and metabolic disorder characterized by reproductive and metabolic abnormality disorders. Dietary factors influence the body composition and hydration status, which may exacerbate PCOS symptoms. The aim of this study was to assess the associations between the habitual nutrient intake and bioelectrical impedance analysis parameters in Polish women with PCOS and healthy controls, in order to identify potential nutritional targets for a non-pharmacological intervention. **Methods:** This study involved 50 women aged 18–45 years (25 with PCOS and 25 healthy). Participants kept 7-day food diaries and their body composition was assessed using the SECA mBCA 515 analyzer. The nutrient intake was compared with EFSA recommendations. **Results:** Women with PCOS had a higher body weight, waist circumference and body mass index, visceral adipose tissue, and fat mass index, despite no difference in their total energy intake. They consumed more omega-3 fatty acids (EPA + DHA) than the control group. Vitamin D deficiency and irregular supplementation were common in both groups. Body composition parameters such as the phase angle and ECW/TBW ratio correlated with the diet quality—especially with protein; fiber; and vitamin B2, B12, and folate levels. **Conclusions:** The obtained results showed significant differences in body compositions and the presence of a relationship between the nutrient intake and bioimpedance parameters in women with PCOS. These results emphasize the importance of a comprehensive nutritional and body composition assessment in planning dietary interventions in this group of patients.

## 1. Introduction

Polycystic ovary syndrome (PCOS) is a complex, multifactorial endocrine disorder influenced by genetic, environmental, dietary, and lifestyle factors. It is estimated to affect between 5% and 15% of premenopausal women worldwide and is considered a leading cause of female infertility [1]. PCOS is a heterogeneous disorder that has a variety of causes and symptoms. Environmental factors—e.g., socioeconomic, geographic, toxicological, lifestyle, and diet-related—and genetic factors—e.g., gene variants, epigenetic, and race/ethnicity—are cited in its formation [2]. Among the most common symptoms of PCOS are irregular menstrual cycles (oligomenorrhea) associated with infertility, a polycystic ovarian morphology on ultrasound, and biochemical and/or chemical features of hyperandrogenism, such as hirsutism, acne, or alopecia areata [3]. Moreover, PCOS is associated with several metabolic disorders, including obesity, hyperinsulinemia, insulin resistance, metabolic syndrome, hepatic steatosis, and dyslipidemia [4].

The lifestyle and dietary behaviors of women are increasingly recognized as crucial modifiable factors influencing both the onset and clinical course of polycystic ovary syndrome (PCOS). While the exact etiology of PCOS is multifactorial and includes genetic, hormonal, and environmental components, a growing body of evidence highlights the central role of nutrition in modulating its metabolic and reproductive manifestations. Obesity, particularly central adiposity, is prevalent among women with PCOS and exacerbates insulin resistance, a key mechanism underlying hyperandrogenism and menstrual dysfunction. Importantly, even in women with a normal body weight, a poor dietary quality can contribute to metabolic disturbances associated with PCOS [5]. Diet plays a pivotal role in regulating insulin sensitivity, inflammation, lipid metabolism, and hormonal balance—all of which are impaired in PCOS. Dietary patterns characterized by the excessive consumption of simple sugars, high-glycemic index (GI) foods, and processed carbohydrates have been associated with elevated postprandial glucose and insulin levels, promoting hyperinsulinemia and insulin resistance. These mechanisms are central to the development of the androgen excess typical in PCOS, as hyperinsulinemia directly stimulates ovarian theca cells to produce androgens while suppressing the hepatic production of sex hormone-binding globulin (SHBG), thereby increasing the bioavailability of circulating androgens [6,7]. Furthermore, the inadequate intake of dietary fiber and essential micronutrients, such as magnesium, zinc, vitamin D, and B-vitamins, may further compromise metabolic flexibility and hormonal regulation. Diets low in omega-3 fatty acids—particularly eicosapentaenoic acid (EPA) and docosahexaenoic acid (DHA)—but high in saturated fatty acids and trans fats can worsen systemic inflammation and negatively impact lipid profiles, further contributing to the metabolic burden in women with PCOS [5].

The literature emphasizes that the diet and nutritional status play an important role in modulating the course and severity of PCOS symptoms [7]. However, most studies in this regard have been conducted mainly in Asian [8], American [9], or Western European [10] populations, where genetic, cultural, and nutritional conditions differ significantly from those in Poland. In the Polish population, there is insufficient data on both the nutrient intake and nutritional status of women with PCOS in the context of their clinical symptoms. Thus, there is a research gap that makes it difficult to adapt dietary recommendations to the specific nutrition of women in Poland.

Polish dietary habits are characterized by a relatively high consumption of animal products, including red and processed meat, and a limited intake of oily fish and fortified foods, which may contribute to a higher intake of saturated fats and a lower intake of vitamin D. Compared to Mediterranean or Nordic dietary patterns, which are often associated with cardiometabolic benefits, the traditional Polish diet may increase the risk of metabolic disturbances [11]. These differences underscore the importance of investigating dietary and metabolic interactions in women with PCOS within the Polish population.

The aim of this study was to assess the dietary intake and nutritional status (with particular emphasis on body composition) in women with PCOS in a Polish population and to analyze their association with clinical manifestations of the syndrome. The results obtained may provide a basis for the development of more personalized dietary recommendations for women with PCOS in Poland.

## 2. Materials and Methods

This study included 50 women aged 18 to 50 years. The study group consisted of 25 women previously diagnosed with polycystic ovary syndrome, while the control group included 25 healthy women, whose results served as a reference point. The diagnosis of PCOS had been previously confirmed by medical specialists based on the Rotterdam criteria, which require the presence of at least two out of three diagnostic features: irregular menstruation or anovulation, clinical and/or biochemical hyperandrogenism, and polycystic ovarian morphology on ultrasound examination [12]. The control group included women with regular menstrual cycles, no symptoms of PCOS, and no reported hormonal disorders. Participants were recruited through social media platforms, announcements posted at the University, and in medical facilities.

During the visit, participants were assigned to groups designated as the study group (women with PCOS) and the control group (healthy women).

Before participating in the study, all women were informed about the purpose of the research, the safety procedures, and the protection of their personal data. They provided written informed consent before enrollment.

This study was conducted in accordance with the Declaration of Helsinki and was approved by the University Ethics Committee for Research Involving Human Subjects (University of Life Sciences in Lublin) on 18 October 2023, approval no. UKE/10/23. All data were fully anonymized prior to access by the authors.

*Exclusion Criteria*

Participants were excluded from the study if they had:

- Other endocrine disorders (e.g., hypothyroidism, hyperprolactinemia);
- Hormone therapy within the last six months;
- Metabolic diseases, such as type 2 diabetes.

Additionally, due to the use of the BIA method for body composition assessment, women meeting any of the following conditions were also excluded:

- Pregnancy;
- Pacemaker implantation;
- Electrodes, stimulators, or other electronic implants;
- Use of diuretics;
- Epilepsy.

### 2.1. Body Composition Analysis

As part of this study, participants visited the Dietitian Service (University of Life Sciences in Lublin, Poland), where body composition was analyzed using bioelectrical impedance analysis (BIA). Bioelectrical impedance analysis (BIA) measurements were performed using the SECA mBCA 515 analyzer (SECA GmbH & Co. KG, Hamburg, Germany) in accordance with the manufacturer’s guidelines and under standardized measurement conditions. The device employs a multi-frequency technology with a tetra-polar electrode placement on the hand and foot, allowing assessment of total and segmental body composition. Participants were asked to fast for at least 4 h before the assessment and to maintain proper hydration while avoiding excessive fluid intake. Measurements were conducted in the morning hours (between 7:00 AM and 10:00 AM) to minimize the effects of diurnal variations in body water content. Additionally, participants refrained from vigorous physical activity for 12 h prior to the measurement and emptied their bladder immediately before the assessment. Measurements were taken in a standing position, with participants adhering to clothing requirements and removing all metal accessories.

### 2.2. Dietary Records

Additionally, dietary habits were assessed based on 7-day dietary records kept by each participant. To enhance the accuracy of the 7-day food diaries, participants received detailed instructions on how to record their food intake, including guidance on estimating portion sizes using household measures. Additionally, all diaries were reviewed by a qualified dietitian (K.N), and when inconsistencies or unclear entries were identified, participants were contacted for clarification via follow-up interviews (in person or by phone). Visual aids illustrating standard portion sizes were also provided to help participants more accurately estimate the quantity of food consumed. While photographic documentation was not used in this study, the combined use of standardized guidance and professional verification aimed to ensure the reliability of the collected dietary data. Additionally, participants were asked to record supplementation in a food diary. Participants were asked to report the types, frequency, and dosage of all supplements taken during the study period. Supplement intake data were analyzed to identify irregular supplementation patterns and were included in the calculation of total nutrient intake by converting supplement doses to nutrient equivalents. This allowed for a comprehensive assessment of overall nutrient intake, taking into account both dietary and supplement sources. To ensure the reliability of the energy intake data, preliminary filtering of caloric values was performed. Recorded daily energy intake below 500 kcal or above 3500 kcal was considered implausible and excluded from further analysis. As a result, only data falling within the range considered reliable were included in the analysis, which allowed us to reduce the influence of errors related to underestimation or overestimation of energy intake and increased the internal validity of the study.

The nutritional value of the consumed foods and meals was calculated using the commercial software Kcalmar.pro (Hermax, Lublin, Poland, https://kcalmar.com/dietetyk/, accessed on 12 January 2025), in accordance with dietary guidelines and specific nutritional recommendations for women with PCOS. Dietary intake data recorded by the participants were processed using the Kcalmar.pro software under the supervision of a certified dietitian (K.N.). The collected data enabled an assessment of energy intake, macronutrients (protein, fat, carbohydrates), minerals, and vitamins. The results from the dietary records were compared with the dietary reference values recommended by the European Food Safety Authority (EFSA) [13].

### 2.3. Statistical Analysis

Statistical analysis of the collected dietary data was performed using Statistica 13.3. To determine differences between groups, Student’s *t*-test was applied for normally distributed data, while the Mann–Whitney U test was used in cases where the distribution was not normal. The normality of distribution was assessed using the Shapiro–Wilk test. Correlations between nutrient intake and body composition parameters were analyzed using Pearson’s correlation coefficient for variables with normal distribution, and Spearman’s rank correlation coefficient was used when at least one variable did not meet the assumption of normality. To minimize the risk of Type I errors, the False Discovery Rate (Benjamini–Hochberg correction) was applied. Additionally, differences in mean values between groups were calculated along with 95% confidence intervals (95% CIs), which allowed for the assessment of the precision and clinical significance of the observed differences. Results were considered statistically significant at *p* < 0.05.

## 3. Results

### 3.1. Characteristics of Groups

The characteristics of the women with PCOS and the control group are shown in Table 1. The comparative analysis between the PCOS group and the control group revealed significant differences in several anthropometric and body composition parameters. The mean body weight was higher by 10.5 kg in the PCOS group; however, the 95% confidence interval (−1.6 to 22.6) indicates some uncertainty around this difference, despite the statistically significant *p*-value (*p* = 0.006). The BMI was significantly higher in the PCOS group by 5.6 kg/m^2^ (95% CI: 2.4 to 8.8), confirming a clear tendency toward overweight and obesity in this population. The fat mass index (FMI) and visceral adipose tissue (VAT) volume were also significantly elevated in women with PCOS, with differences of 4.5 kg/m^2^ (95% CI: 2.0 to 7.0) and 0.9 L (95% CI: 0.27 to 1.53), respectively. The waist circumference was 13.5 cm greater (95% CI: 5.6 to 21.4) in the PCOS group, highlighting an increased risk of metabolic complications related to central obesity. It is worth noting that the physical activity level (PAL) was slightly but significantly lower in the PCOS group (−0.1; 95% CI: −0.18 to −0.02). The skeletal muscle mass (SMM) and fat-free mass index (FFMI) were higher in the PCOS group, which may reflect metabolic adaptations related to an increased body mass. The decreased resistance value by 66 Ω (95% CI: −118 to −14) in the PCOS group might indicate an altered body water composition, although differences in the reactance and phase angle were not statistically significant.

### 3.2. Analysis of Nutrient Intake

In the dietary record analysis, the energy intake, macronutrient consumption, and selected nutrients (saturated fatty acids, omega-3 fatty acids, and fiber) were compared between women with PCOS and the control group (Table 2). The average energy intake was higher in the PCOS group (1909 ± 404.7 kcal) compared to controls (1741 ± 254.3 kcal), with a mean difference of 168 kcal (95% CI: −16 to 352), but this difference was not statistically significant (*p* = 0.086).

Regarding the macronutrient intake, the mean values for protein, carbohydrates, and fat in the PCOS group were 87.8 ± 17.6 g, 231.0 ± 66.8 g, and 71.1 ± 15.8 g, respectively, while in the control group, the corresponding values were 81.9 ± 15.3 g, 197.7 ± 34.7 g, and 64.8 ± 17.8 g. Similarly, no significant differences were found in the protein (mean difference 5.9 g; 95% CI: −5.1 to 16.9; *p* = 0.362), carbohydrates (34 g; 95% CI: −14 to 82; *p* = 0.497), and fat (6.3 g; 95% CI: −6.4 to 19.0; *p* = 0.555). The saturated fatty acid (SFA) intake was similar in both groups, with 22.4 ± 6.8 g in the PCOS group and 19.4 ± 6.6 g in the control group (3.0 g; 95% CI: −3.4 to 9.4; *p* = 0.818), indicating no significant differences. However, a statistically significant difference (95% CI: 46 to 558; *p* = 0.023) was observed for the omega-3 fatty acid intake. Women with PCOS consumed an average of 452.2 ± 497.8 mg of omega-3 fatty acids, whereas the control group had an average intake of 150.8 ± 198.8 mg.

The analysis of the vitamin intake showed no statistically significant differences between women with PCOS and controls across all measured vitamins (B1, B2, B3, B6, folic acid, B12, C, A, D, and E) (Table 3). Mean differences between groups were small and their 95% confidence intervals included zero, indicating an uncertainty regarding any true difference in vitamin consumption.

For example, the vitamin B1 intake was slightly higher in the PCOS group by 0.3 mg (95% CI: −0.1 to 0.7; *p* = 0.562), while the vitamin B12 intake was marginally lower by 0.3 µg (95% CI: −1.1 to 0.5; *p* = 0.631) compared to controls. Similarly, vitamins C and E showed a higher mean intake in PCOS, but without a statistical significance.

The average intake of individual minerals in women with PCOS and the control group is presented in Table 4. The analysis of the mineral intake showed no statistically significant differences between women with PCOS and controls across all measured minerals, including sodium, potassium, calcium, phosphorus, magnesium, iron, zinc, copper, iodine, and manganese. The mean differences were generally small, and the 95% confidence intervals included zero, indicating no clear evidence of differences in the mineral consumption between the two groups.

For instance, the potassium intake was higher in the PCOS group by 408 mg (95% CI: −25 to 841; *p* = 0.065), approaching but not reaching statistical significance. Similarly, the copper intake was slightly higher in the PCOS group by 0.3 mg (95% CI: −0.1 to 0.7; *p* = 0.215).

The following section presents the results shown in the Supplementary Tables (Tables S1–S6). In the study group, the average energy intake was 1068–2692 kcal per day (Table S1). The estimated energy requirement of 1820 to 2768 kcal covered the needs of the majority of the study participants, and only 6 out of 25 participants had less than 80% of this requirement covered. The consumption of macronutrients in the study group was most often in line with the recommendations, although deviations were observed in several cases. Protein constituted 12 to 25% and carbohydrates 39 to 61% of the energy from the diet, 56–132 and 108–386 g, respectively. Fats and SFAs constituted 26–41 and 8–18% of the energy, respectively, with more than the recommended 35% of energy from fats and 10% from saturated fatty acids noted in 9 and 13 participants, respectively. The fiber intake was on average 24.4 g, with 13 participants consuming less than 25 g and 7 less than 20 g. Similarly to the control group, the eicosapentaenoic acid (EPA) and docosahexaenoic acid (DHA) intake was very variable and ranged from 0 to 1720 mg/day, with an intake above 250 mg/day recorded in only 10 participants.

The analysis of dietary records in the control group revealed that the average daily energy intake ranged from 1790 to 2120 kcal, with individual values varying significantly (Table S2). The estimated energy requirement (EER) for participants ranged from 1623 to 2850 kcal, indicating that some women consumed less than their theoretical energy needs, while others exceeded the reference values.

The macronutrient intake in the control group aligned with dietary recommendations, although a considerable individual variability was observed. The average protein intake ranged from 50 to 107 g/day, accounting for 15–23% of the total energy intake, and, in most cases, met the Population Reference Intake (PRI) recommendations. The fat intake ranged from 136 to 278 g/day (30–40% of total energy intake), while the SFA consumption varied between 6.9 and 38.8 g/day, highlighting significant dietary differences among participants. The carbohydrate intake ranged from 145 to 289 g/day (42–55% of total energy intake), which was consistent with the recommended range for adult women.

The vitamin intake in the study group was in most cases in line with the standards (Table S3). All individuals in the study group met the recommendations for thiamine, and in the case of niacin and vitamin B6, C, and A, the recommended intake was not met by only a few individuals. In the case of riboflavin (consumption from 0.9 to 3.2 mg), the average requirement (AR) standard was not met by every fourth participant, and the PRI standard was met by almost half of the subjects. The situation was similar in the case of folic acid (consumption from 128 to 712 μg Dietary Folate Equivalent (DFE)), where a deficiency in the AR standard was found in four participants, and the PRI standard was not met by 11 subjects. In the case of vitamin B12, only every fifth subject met the EFSA standards. However, in Poland, the recommended dietary allowance (RDA) standard for cobalamin is 2.4 μg/day [14], and from this perspective, over 70% consumed the appropriate amount of this vitamin. Similarly to the control group, an insufficient vitamin D intake was found in all study participants. In the diets analyzed, its presence was found at the level of 1.1–7.5 µg/day, which of course clearly indicates the need for supplementation. The intake of vitamin E in most women in the study group did not meet the standards; at the same time only seven noted an intake below an 80% adequate intake (AI).

The vitamin intake in the control group generally met or exceeded reference standards, especially for B vitamins and vitamin C and A (Table S4). The thiamine intake ranged from 0.5 to 1.8 mg/day (78–334% of AR), riboflavin from 1.0 to 2.1 mg/day (77–162% AR), niacin from 9.9 to 22.3 mg/day (100–299% AR), and pyridoxine from 1.1 to 2.4 mg/day (85–185% AR). The folate intake varied widely (164–522 µg/day, 65–285% AR), with some participants below recommended levels, suggesting a deficiency risk. Vitamin B12 ranged from 1.5 to 6.6 µg/day, with a few near the lower limit (38–165% AI). The vitamin D intake was low (0.8–7.4 µg/day, 5–49% AI), indicating a need for supplementation. The vitamin E intake ranged from 4.5 to 13.4 mg/day (41–122% AI), with some participants below recommended levels.

In the study group, the phosphorus intake was sufficient for all participants, and the zinc intake was adequate in over 80% of cases (Table S5). However, deficiencies were common for iodine, calcium, and iron. None of the women met iodine requirements, with intakes ranging from 6 to 62% of the AI. The calcium intake was low, with only two women meeting standards (250–1417 mg/day). The iron intake varied (6.2–19.7 mg/day), but 72% did not meet the PRI, despite most meeting the AR. The sodium and potassium intakes showed variability but were mostly adequate. The copper intake was insufficient in about half of participants. Magnesium and manganese deficiencies affected up to 40% of the group, though applying an 80% AI threshold reduced this to around 20%.

In the control group, the mineral intake generally aligned with recommendations (Table S6), but some participants showed low intakes of calcium, iron, iodine, and copper. The phosphorus intake was consistently adequate. Variability in sodium, potassium, magnesium, zinc, and manganese was observed, with some individuals at risk of deficiency.

### 3.3. The Analysis of the Correlation Between the Nutrient Intake and Body Composition in Women with PCOS

**Figure 1:**
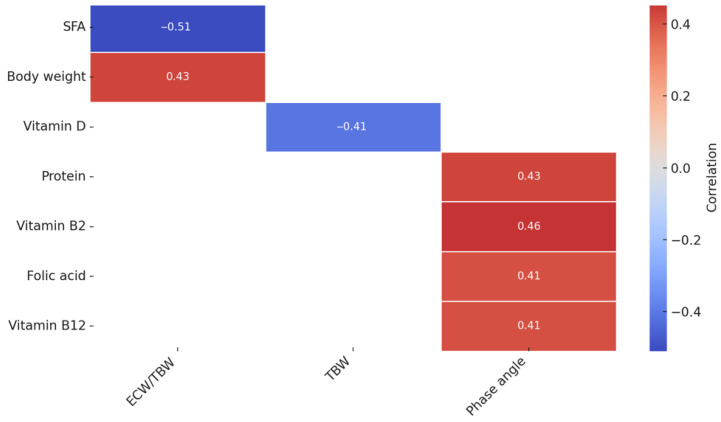
A heatmap of significant correlations between the nutrient intake and body composition analysis results in the study group (with PCOS). Colors indicate the strength and direction of correlations: shades of red indicate a positive correlation, while shades of blue indicate a negative correlation. The more intense the color, the stronger the relationship. Correlations are statistically significant (*p* < 0.05). TBW—total body water, ECW—extracellular water, and SFAs—saturated fatty acids.

In the analyzed group of women with PCOS, several significant relationships were observed between body composition parameters and selected nutrients (Figure 1). A positive correlation was found between the body weight and the extracellular water to the total body water ratio (ECW/TBW ratio) (r = 0.43). In addition, saturated fatty acids showed a negative correlation with the ECW/TBW (r = −0.51), and the total body water (TBW) was significantly inversely correlated with vitamin D levels (r = −0.41). The phase angle, a marker of the cell membrane integrity and general nutritional status, correlated positively with protein levels (r = 0.43), vitamin B2 (r = 0.46), folic acid (r = 0.41), and vitamin B12 (r = 0.41).

**Figure 2:**
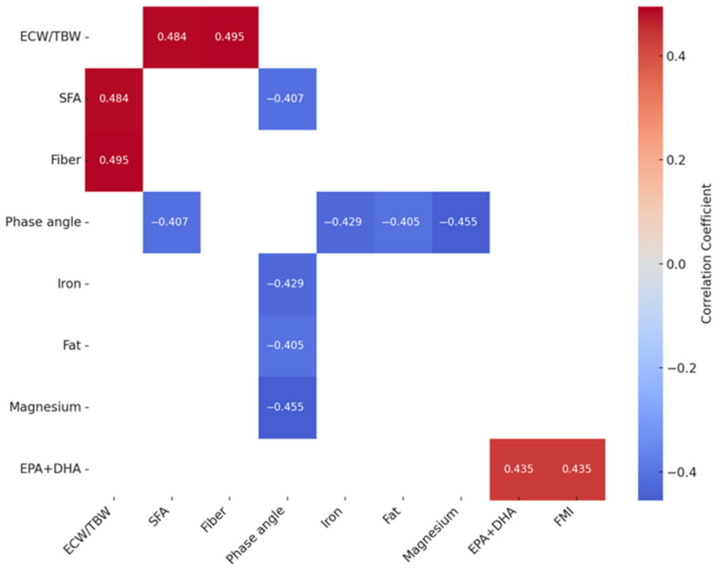
A heatmap of significant correlations between nutrient intake and body composition analysis results in the control group (without PCOS). Colors indicate the strength and direction of correlations: shades of red indicate a positive correlation, while shades of blue indicate a negative correlation. The more intense the color, the stronger the relationship. Correlations are statistically significant (*p* < 0.05). TBW—total body water, ECW—extracellular water, SFAs—saturated fatty acids, EPA—eicosapentaenoic acid, DHA—docosahexaenoic acid, and FMI—fat mass index.

In the group of women without PCOS (Figure 2), several significant associations were also observed between body composition parameters and selected dietary components. The ECW/TBW ratio showed a positive correlation with the intake of saturated fatty acids (r = 0.484) and dietary fiber (r = 0.495). The phase angle was negatively correlated with SFA levels (r = −0.407), as well as with iron (r = −0.429) and magnesium intakes (r = −0.455). Additionally, the total fat intake also showed a negative relationship with the phase angle (r = −0.405). Furthermore, the EPA + DHA fatty acid intake was positively correlated with the FMI (r = 0.435).

## 4. Discussion

This study examined the relationship between the dietary intake and body composition in women with PCOS compared to healthy controls, aiming to explore how nutritional factors and the nutritional status may influence the clinical presentation of PCOS.

### 4.1. Body Composition Analysis

In the presented study, women with PCOS were characterized by a significantly higher body mass, BMI, FMI, VAT, and waist circumference compared to the control group. These results are consistent with previous reports, which indicate that excess body mass and abdominal obesity are common clinical features in women with PCOS and are associated with an increased risk of insulin resistance, metabolic disorders, and cardiovascular complications [15,16]. Central obesity and increased visceral fat accumulation play a key role in the pathophysiology of the syndrome, exacerbating chronic inflammation and hormonal disorders [17].

The lean mass and skeletal muscle mass indices did not differ significantly between the groups, which may indicate that the main differences in the body composition of women with PCOS concern the fat component, not the lean body mass. In the studied group of women with PCOS, a higher FMI was also noted, which is increasingly seen as a more precise parameter for assessing obesity than the BMI alone, because it takes into account the actual share of adipose tissue in relation to growth [18]. An increased FMI may therefore be a useful marker of metabolic risk in the PCOS population [19].

It is worth noting that this study did not show any differences in heights or physical activity levels, which suggests that the observed differences in the body composition are mainly due to metabolic factors and possible differences in dietary habits.

### 4.2. Dietary Intake Assessment

Women with PCOS exhibited a higher caloric intake. This may be linked to the quality of food choices, which likely contributed to the greater body weight, waist circumference, and fat mass index observed in the PCOS group (all statistically significant differences). Similar findings were reported by Douglas et al. [20], who noted that women with PCOS tend to consume more high-glycemic index foods such as white bread, refined pasta, sweets, and fruits compared to healthy controls, potentially elevating fasting insulin and glucose levels. Such dietary patterns may exacerbate insulin resistance and other metabolic complications in PCOS [21]. Furthermore, central obesity, often seen in women with PCOS, is closely associated with an increased risk of metabolic disorders, such as insulin resistance, type 2 diabetes, and cardiovascular diseases [22]. Therefore, a low-glycemic index (GI) diet may be especially beneficial for women with PCOS. Barr et al. [23] demonstrated that a low-GI diet improved insulin sensitivity in women with PCOS over a 12-week intervention, independent of weight change.

The individual analysis of food diaries revealed a variability in the energy intake among participants—some consumed less than their estimated requirements, while others exceeded them. This variation may reflect differences in lifestyles, physical activity, or attempts to modify diets following a PCOS diagnosis. Many studies based on the analysis of food diaries also show that the energy intake is commonly underestimated, with discrepancies reaching up to 12% [24,25]. Carbohydrates contributed 42–55% of the total energy intake in both groups. Although the mean carbohydrate intake in our PCOS group was higher than in the control group, the analysis of individual diaries showed that some women followed a diet with a reduced carbohydrate intake. A lower carbohydrate intake—particularly when combined with a low glycemic index—has been associated with improvements in insulin resistance, reductions in circulating androgen levels, and better ovulatory function [26]. However, it is also important to note that excessively reducing the carbohydrate intake—particularly when it falls below recommended levels or is not properly balanced with an adequate fiber and micronutrient intake—may carry certain risks. Very-low-carbohydrate diets can lead to a reduced intake of dietary fiber, B vitamins, and certain antioxidants, potentially affecting gut health, energy metabolism, and the overall diet quality [27].

The protein intake also varied, but most participants met the PRI recommendations. Nevertheless, some individuals may still consume suboptimal amounts. Adequate protein supports fat loss and lean mass preservation, which is particularly relevant in PCOS due to its importance for metabolic function [28].

The fat intake was higher among women with PCOS, including both saturated fats and omega-3 fatty acids. This indicates that some individuals adhered to healthier dietary patterns, while others exceeded the recommended saturated fat intake—potentially worsening PCOS symptoms, such as insulin resistance and elevated androgen levels [29]. Interestingly, women with PCOS in this study consumed significantly more omega-3s (EPA + DHA) than controls. Supplementation with omega-3 fatty acids has been shown to improve insulin sensitivity and reduce insulin resistance in women with PCOS [30,31]. Additionally, their anti-inflammatory effects (e.g., reducing C-reactive protein (CRP) levels) may help alleviate hyperandrogenic symptoms [32]. Given the role of chronic inflammation in the pathophysiology of PCOS, dietary choices high in saturated fats and high-glycemic index foods may further exacerbate inflammatory processes. In turn, an increased fiber and omega-3 fatty acid intake may have a protective effect by, among other things, reducing CRP levels and improving insulin sensitivity [33].

The fiber intake met recommended levels (25 g/day) in most participants. A higher fiber intake in women with PCOS is associated with better insulin sensitivity and is inversely correlated with the homeostatic model assessment of insulin resistance (HOMA-IR) and abdominal fat [34]. Acharya et al. [35] found that women with PCOS consume less fiber, particularly soluble fractions, than women without PCOS.

A non-significant trend toward a higher potassium intake was observed in the PCOS group. Potassium plays a role in the fluid–electrolyte balance, acid–base homeostasis, fatty acid metabolism, and inflammation modulation [36]. Diets high in potassium are associated with a reduced PCOS risk due to anti-inflammatory effects [37].

The vitamin D intake was insufficient across participants, likely due to limited dietary sources. Sunlight-induced skin synthesis remains the primary determinant of the vitamin D status, but the seasonal variation, latitude, and lifestyle factors (e.g., indoor activity, sunscreen use) further contribute to deficiency, especially in higher latitudes [38]. Supplementation is thus necessary, particularly in women with PCOS [39,40]. However, participants in this study did not report consistent supplementation.

The results presented by Douglas et al. [9], who analyzed the dietary diaries of female college students struggling with PCOS in the United States, showed that the average energy intake in the study group was approximately 1759 kcal/day. This value is similar to the average energy intake in the control group without PCOS in our study, while the women with PCOS in our study group were characterized by a higher energy intake. In the study by Douglas et al. [9], a lower intake of carbohydrates and fiber was also found. The common result of both studies was an increased intake of saturated fats.

In the case of the study by Navarro-Lafuente et al. [10], significant differences were found between the diet of women with PCOS and women without PCOS from Spain. Women with PCOS had a higher BMI than women in the control group, which was identical to the results of our study. In this study, a similar energy intake was found in both groups, while the intake of carbohydrates, protein, fat, and fiber did not differ significantly between the groups, which was also shown in our study. There were also no significant differences in the intake of omega-3 fatty acids between women with PCOS and the control group. The omega-3 intake in both groups was low and similar (~1.6 g/day).

In another study comparing women with PCOS and healthy control women from Italy, no significant differences were found in the total energy, protein, carbohydrate, and total fat intake between the analyzed groups. However, women with PCOS had a higher fiber intake compared to the control group and a lower percentage of energy from fat. Some differences were also observed in dietary preferences—women with PCOS more often consumed sweets, cheese, and larger amounts of oils. The consumption of vegetables, fruits, meat, and legumes was similar in both groups. Additionally, no significant differences were found in the consumption of advanced glycation end products (AGEs) between women with PCOS and the control group [41].

### 4.3. Associations Between Dietary Intake and Body Composition Parameters

In recent years, there has been growing interest in analyzing the relationship between the diet and body composition in women with PCOS. Diets can affect not only body weight or insulin sensitivity, but also bioimpedance parameters that reflect the state of cell membranes and water and electrolyte management [42].

Observed correlations between body composition parameters and selected nutrients highlight potential diet–metabolism interactions in women with PCOS. The positive association between the body weight and ECW/TBW ratio may indicate greater fluid retention in individuals with excess weight—a common trait in PCOS metabolic phenotypes. Elevated extracellular water may also reflect chronic inflammation and metabolic dysfunction associated with the syndrome [43].

The phase angle, an indicator of cellular membrane function, showed positive correlations with protein and B vitamins (B2, folate, B12). These findings align with previous research emphasizing the role of B vitamins in energy metabolism, cellular proliferation, and antioxidant defense [39]. An adequate intake may thus support the membrane integrity and overall nutritional status [44]. Women with PCOS often have deficiencies of B vitamins, which, through their participation in methylation pathways and the regulation of the homocysteine cycle, may additionally affect the fat and glucose metabolism and body weight [45]. B vitamins, especially B2 (riboflavin), B6 (pyridoxine), B9 (folate), and B12 (cobalamin), play a key role as coenzymes in numerous metabolic pathways, including the one-carbon cycle and the methylation cycle. The proper activity of these pathways determines the maintenance of homocysteine homeostasis, an amino acid whose elevated levels are often observed in women with PCOS and are associated with a higher risk of metabolic and cardiovascular complications [46].

The phase angle is indirectly related to the oxidative status, and an oxidative–antioxidant imbalance plays an important role in the pathophysiology of PCOS by reflecting the integrity of cell membranes susceptible to oxidative damage [47].

The vitamin D intake showed a negative correlation with the total body water, which may reflect an impaired hydration status and increased extracellular water in vitamin-D-deficient individuals—which is common in women with PCOS [48]. In the control group, the phase angle correlated negatively with saturated fat, iron, and magnesium, while the EPA + DHA intake showed a positive association with the FMI (r = 0.44). These associations may further support the relevance of the fat quality and micronutrient intake in shaping metabolic profiles [49].

This study highlights the value of personalized nutrition in PCOS management. The adequate intake of B vitamins (B2, B12, folic acid) and protein may improve cellular health, as reflected by higher phase angle values. Limiting saturated fat and increasing the omega-3 intake may support better hydration and metabolic balance. Given the common vitamin D deficiency, regular supplementation is advisable. Including a bioimpedance analysis in dietary monitoring could help tailor interventions to individual needs and track improvements in the nutritional status.

### 4.4. Study Limitations and Strengths

This study has several limitations that should be considered when interpreting the results. First, the relatively small sample size limits the statistical power and generalizability of the findings, particularly for subgroup or mediation analyses. Nevertheless, the data provide valuable preliminary insights and may inform the design of future, larger-scale studies. Second, the dietary intake was assessed using self-reported methods, which are prone to recall bias and under- or over-reporting. However, the use of a standardized and validated dietary assessment tool ensured methodological consistency and practical applicability for population-based research. Third, the cross-sectional design prevents us from establishing causal relationships between dietary factors, body compositions, and metabolic parameters. Still, it allows for the identification of current associations and potential risk patterns that warrant further longitudinal investigation. Lastly, the participant recruitment through social media and university posters may have resulted in a sample that is not fully representative of the general female population in Poland. At the same time, this approach enabled efficient access to a target group—young women with an interest in health or PCOS—which enhances the contextual relevance of the findings.

While the research question may have a limited novelty, this work addresses a gap in the regional literature and provides initial, exploratory data from a population that is often underrepresented in PCOS research. The use of indirect methods to assess the body composition, such as bioelectrical impedance analysis (BIA), may also be viewed as a limitation; however, the BIA remains a widely used, non-invasive, and cost-effective tool for estimating body compositions in clinical and epidemiological settings. In this study, we utilized a medical-grade BIA device (SECA mBCA 515) that employs 17 measurement frequencies ranging from 1 kHz to 1000 kHz, allowing for a detailed and segmental analysis of body composition. This advanced technology has been validated against gold-standard methods such as DEXA and supports the reliability of our measurements despite the indirect nature of the BIA.

Given these constraints, the present study should be interpreted as a pilot investigation. Future studies should aim to include a larger and more stratified sample (e.g., by age, socioeconomic background, and PCOS severity) and incorporate additional clinical parameters (e.g., hormonal profiles, laboratory markers) to allow for more robust statistical modeling and broader applicability. Despite its limitations, this study contributes meaningfully to our understanding of dietary variability and bioimpedance profiles in women with PCOS. It highlights the potential value of personalized nutritional guidance and the use of simple, non-invasive tools such as the BIA for assessing the nutritional status and metabolic risk in this population.

## 5. Conclusions and Future Research Directions

Although no statistically significant differences were observed between the PCOS and control groups in the energy or macronutrient intake, a trend toward a higher intake of energy, saturated fat, omega-3 fatty acids, and several micronutrients was noted among women with PCOS. Notably, the omega-3 intake was significantly higher in the PCOS group (*p* = 0.0226), which may reflect an increased nutritional awareness or supplement use. These findings suggest that while general dietary patterns may appear similar across groups, women with PCOS may require more personalized nutritional support.

The high variability observed in the dietary intake among PCOS participants underscores the importance of individualized dietary strategies. Personalized nutritional therapy—tailored to the individual metabolic needs, body composition, and lifestyle—may offer a more effective non-pharmacological management of PCOS, especially given its heterogeneity. Importantly, parameters such as the phase angle and ECW/TBW ratio obtained through the bioelectrical impedance analysis (BIA) may serve as useful, non-invasive biomarkers to monitor nutritional status and early metabolic disturbances.

Our findings highlight a considerable variability in the intake of both macro- and micronutrients among women with PCOS, with some showing dietary patterns that may support improved insulin sensitivity. These results underscore the need for individualized nutritional counseling in this group. Importantly, this study offers practical guidance by identifying common dietary shortcomings—such as an insufficient intake of calcium, iron, and iodine—that should be addressed in nutritional planning.

From a clinical and public health perspective, these findings highlight the potential of using simple, accessible tools such as the bioelectrical impedance analysis (BIA) to enhance dietary counseling and support early risk stratification in women with PCOS. Given the heterogeneity of PCOS and the variability in the dietary intake observed in this study, personalized nutritional strategies tailored to individual metabolic profiles and body compositions may be especially effective. Early, targeted nutritional interventions—based not only on caloric balance but also on the nutrient quality—could play a role in mitigating metabolic and reproductive complications associated with the syndrome. Moreover, our results may serve as a valuable reference point for dietitians, endocrinologists, and primary care providers aiming to implement more precise and proactive dietary approaches in women with PCOS.

Future research should focus on validating these findings in larger and more diverse cohorts using longitudinal or interventional designs to explore causal relationships between the dietary intake, body composition, and metabolic risk. Studies incorporating biochemical markers of the nutrient status, inflammatory load, and insulin sensitivity are particularly warranted. Moreover, evaluating dietary interventions across different PCOS phenotypes may improve our understanding of individualized treatment responses. Integrating nutritional assessments with hormonal and metabolic profiling could ultimately enhance both prevention and personalized management strategies for women affected by PCOS.

## Figures and Tables

**Table 1:** Characteristics of the study and control groups.

Parameter	PCOS (n = 25)	Control (n = 25)	*p*-Value	Mean Difference (95% CI)
Age (years)	29.7 ± 9.0	27.1 ± 7.0	0.344	2.6 (−2.3 to 7.5)
Body weight (kg)	78.3 ± 18.4	67.8 ± 23.8	0.006 *	10.5 (−1.6 to 22.6)
Height (cm)	165 ± 5.6	162 ± 21.6	0.704	3.0 (−6.0 to 12.0)
BMI (kg/m^2^)	28.5 ± 6.1	22.9 ± 4.6	0.001 *	5.6 (2.4 to 8.8) *
PAL	1.4 ± 0.1	1.5 ± 0.1	0.589	−0.1 (−0.18 to −0.02) *
FMI (kg/m^2^)	11.6 ± 4.5	7.1 ± 3.4	0.023 *	4.5 (2.0 to 7.0) *
FFMI (kg/m^2^)	17.0 ± 1.8	15.8 ± 1.5	0.897	1.2 (0.14 to 2.26) *
SMM (kg)	21.7 ± 3.5	19.9 ± 2.2	0.603	1.8 (0.10 to 3.50) *
VAT (L)	1.4 ± 1.1	0.5 ± 0.5	0.001 *	0.9 (0.27 to 1.53) *
Waist circumference (cm)	87.2 ± 16.3	73.7 ± 7	0.003 *	13.5 (5.6 to 21.4) *
Resistance (Ω)	662 ± 91.1	728 ± 90.1	0.638	−66 (−118 to −14) *
Reactance (Ω)	59.0 ± 8.2	63.9 ± 7.7	0.542	−4.9 (−10.3 to 0.5)
Phase angle [°]	5.1 ± 0.3	5.0 ± 0.4	0.857	0.1 (−0.15 to 0.35)

The statistical significance between groups was marked with an asterisk, BMI—Body Mass Index; PAL—Physical Activity Level; FMI—Fat Mass Index; FFMI—Fat-Free Mass Index; SMM—Skeletal Muscle Mass; and VAT—Visceral Adipose Tissue.

**Table 2:** Comparison of selected nutrient intake between groups.

Parameter	PCOS (n = 25)	Control (n = 25)	*p*-Value	Mean Difference (95% CI)
Energy value (kcal)	1909 ± 404	1741 ± 254	0.086	168 (−16 to 352)
Protein (g)	87.8 ± 17.6	81.9 ± 15.3	0.362	5.9 (−5.1 to 16.9)
Carbohydrate (g)	231 ± 66.8	197 ± 34.7	0.497	34 (−14 to 82)
Fat (g)	71.1 ± 15.8	64.8 ± 17.8	0.555	6.3 (−6.4 to 19.0)
SFA (g)	22.4 ± 6.8	19.4 ± 6.6	0.818	3.0 (−3.4 to 9.4)
Omega-3 fatty acids (mg)	452 ± 497	150 ± 198	0.023 *	302 (46 to 558) *
Fiber (g)	24.4 ± 8.3	24.0 ± 6.2	0.689	0.4 (−5.0 to 5.8)

The statistical significance between groups was marked with an asterisk; SFAs—saturated fatty acids.

**Table 3:** Comparison of intake of selected vitamins between groups.

Parameter	PCOS (n = 25)	Control (n = 25)	*p*-Value	Mean Difference (95% CI)
Vitamin B1 (mg)	1.3 ± 0.4	1.0 ± 0.3	0.562	0.3 (−0.1 to 0.7)
Vitamin B2 (mg)	1.7 ± 0.6	1.4 ± 0.4	0.204	0.3 (−0.1 to 0.7)
Vitamin B3 (mg)	20.4 ± 8.2	16.9 ± 3.8	0.787	3.5 (−3.0 to 10.0)
Vitamin B6 (mg)	2.1 ± 0.7	1.8 ± 0.4	0.211	0.3 (−0.1 to 0.7)
Folic acid (µg)	358 ± 127	321 ± 83.3	0.984	37 (−54 to 128)
Vitamin B12 (µg)	3.0 ± 1.0	3.3 ± 1.4	0.631	−0.3 (−1.1 to 0.5)
Vitamin C (mg)	153 ± 62.6	126 ± 54.4	0.741	27 (−33 to 87)
Vitamin A (µg)	1036 ± 451	1060 ± 356	0.653	−24 (−242 to 194)
Vitamin D (µg)	3.1 ± 1.8	2.8 ± 1.7	0.904	0.3 (−0.9 to 1.5)
Vitamin E (mg)	10.7 ± 3.5	8.8 ± 2.5	0.617	1.9 (−0.8 to 4.6)

**Table 4:** Comparison of intake of selected minerals between groups.

Parameter	PCOS (n = 25)	Control (n = 25)	*p*-Value	Mean Difference (95% CI)
Sodium (mg)	1998 ± 443	1862 ± 534	0.330	136 (−100 to 372)
Potassium (mg)	3117 ± 906	2709 ± 592	0.065	408 (−25 to 841)
Calcium (mg)	686 ± 258	660 ± 181	0.865	26 (−72 to 124)
Phosphorus (mg)	1305 ± 359	1199 ± 218	0.347	106 (−74 to 286)
Magnesium (mg)	341 ± 110	331 ± 161	0.865	10 (−64 to 84)
Iron (mg)	13.1 ± 3.7	11.2 ± 2.2	0.936	1.9 (−0.6 to 4.4)
Zinc (mg)	9.5 ± 2.3	9.0 ± 2.2	0.516	0.5 (−0.9 to 1.9)
Copper (mg)	1.4 ± 0.6	1.1 ± 0.3	0.215	0.3 (−0.1 to 0.7)
Iodine (µg)	41.6 ± 19.1	33.8 ± 13.5	0.865	7.8 (−4.0 to 19.6)
Manganese (mg)	3.4 ± 1.2	3.9 ± 1.6	0.441	−0.5 (−1.3 to 0.3)

## Data Availability

The data is contained within the article.

## Supplementary Materials

The following supporting information can be downloaded at https://www.mdpi.com/article/10.3390/nu17142377/s1: Table S1: Assessment of energy and macronutrient intake in subjects with PCOS; Table S2: Assessment of energy and macronutrient intake in subjects without PCOS; Table S3: Dietary intake of selected vitamins in subjects with PCOS; Table S4: Dietary intake of selected vitamins in subjects without PCOS; Table S5: Dietary intake and AR, PRI, and AI coverage (%) of selected minerals in subjects with PCOS; and Table S6: Dietary intake and AR, PRI, and AI coverage (%) of selected minerals in subjects without PCOS. References [13,50] are cited in the supplementary materials.
