# Supplementary material for: Dietary and Genetic Aspects of Polycystic Ovary Syndrome (PCOS) in Polish Women—Part I: Nutritional Status and Dietary Intake
Source: Nutrients. 2025 Jul 21;17(14):2377. doi: 10.3390/nu17142377 (PMC12300548; doi:10.3390/nu17142377)
Supplement: Supplementary file 1 [file nutrients-17-02377-s001.zip › nutrients-3736838-supplementary.pdf]

Table S1. Assessment of energy and macronutrient intake in subjects with PCOS*

No.	Daily energy intake [kcal]	EER	EER (%)	Protein				Fat		SFA		Carbohydrates		Fiber		EPA+DHA	
				Average Intake(g)	E (%)	AR (%)	PRI (%)	Average Intake (g)	E (%)	Average Intake (g)	E (%)	Average Intake (g)	E (%)	Average Intake (g)	AI (%)	Average Intake (g)	AI (%)
1	1584	1915	83	72.4	18	203	162	63.5	36	20.4	12	191	48	22.4	90	250	100
2	2314	1984	117	99.5	17	296	235	98.5	38	27.2	11	231	40	34.1	136	800	320
3	2121	1941	109	100	19	273	217	79.8	34	29.9	13	264	50	26.9	108	0	0
4	1945	2149	91	71.4	15	164	130	59.8	28	21.1	10	268	55	26.3	105	300	120
5	1303	2296	57	66.1	20	171	136	54.5	38	14.4	10	199	61	18.1	72	1100	440
6	1749	2768	63	89.2	20	214	170	58.8	30	16.6	9	226	52	19.8	79	1700	680
7	1911	2751	69	88.0	18	225	179	87.2	41	21.3	10	186	39	22.4	90	200	80
8	1327	1820	73	80.4	24	221	176	49.6	34	12.3	8	134	41	25.1	100	1200	480
9	1574	2363	67	60.2	15	150	119	70.2	40	14.2	8	192	49	11.4	46	250	100
10	2692	2528	106	132	20	324	258	99.4	33	32.3	11	322	48	24.1	96	330	132
11	1895	2237	85	88.8	19	237	188	60.2	29	20.1	10	220	47	27.8	111	250	100
12	2004	1831	109	111	22	293	233	75.4	34	39.6	18	214	43	21.1	84	700	280
13	1515	1844	82	95.4	25	246	196	49.8	30	17.5	10	162	43	9.24	37	160	64
14	2292	2050	112	92.5	16	207	164	66.0	26	33.2	13	341	60	29.4	118	770	308
15	2280	2225	102	98.8	17	253	201	86.1	34	24.3	10	302	53	34.2	137	150	60
16	2610	2350	111	102	16	279	222	80.4	28	23.4	8	385	59	28.4	114	160	64
17	1800	2034	88	96.7	21	287	228	66.7	33	17.8	9	192	43	23.1	92	500	200
18	1068	2500	43	63.0	24	155	123	44.5	38	13.7	12	108	41	14.7	59	100	40
19	1957	1884	104	95.2	19	264	210	87.4	40	29.1	13	188	39	31.8	127	100	40
20	1764	2023	87	99.4	23	271	216	66.4	34	25.1	13	202	46	20.1	80	80	32
21	1925	2179	88	81.7	17	223	177	70.0	33	16.7	8	261	54	37.3	149	160	64
22	1761	1926	91	67.4	15	163	130	61.4	31	20.1	10	191	43	19.2	77	125	50
23	1850	2287	81	56.1	12	148	118	72.3	35	21.5	10	217	47	13.2	53	100	40
24	2580	2114	122	97.8	15	260	207	101	36	26.9	9	343	53	44.7	179	1720	688
25	1923	2165	89	89.0	19	226	179	67.7	32	20.1	9	225	47	26.0	104	100	40

*Population reference intakes (PRIs), the average requirements (ARs), and adequate intakes (AIs) were taken from Dietary Reference Values for nutrients Summary report by European Food Safety Authority (EFSA) [13]; EER - Estimated Energy Requirement; SFA - saturated fatty acids, EPA - eicosapentaenoic acid, DHA - docosahexaenoic acid

Table S2. Assessment of energy and macronutrient intake in subjects without PCOS*

No.	Daily energy intake [kcal]	EER	EER (%)	Protein				Fat		SFA		Carbohydrates		Fiber		EPA+DHA	
				Average Intake (g)	E (%)	AR (%)	PRI (%)	Average Intake (g)	E (%)	Average Intake (g)	E (%)	Average Intake (g)	E (%)	Average Intake (g)	AI (%)	Average Intake (g)	AI (%)
1	1790	2227	80	78.4	18	200	159	72.9	37	25.9	13	194	43	24.1	96	0	0
2	1834	2272	81	88.5	19	226	180	63.8	31	22.1	11	211	46	26.5	106	0	0
3	2018	2312	87	92.8	18	247	196	89.1	40	24.6	11	191	38	28.8	115	140	56
4	1558	2048	76	67.1	17	180	143	59.5	34	18.3	11	172	44	26.3	105	0	0
5	1629	1986	82	72.3	18	183	145	54.3	30	12.1	7	217	53	23.9	96	146	58
6	1518	1882	81	66.2	17	158	126	53.0	31	14.9	9	181	48	25.4	102	0	0
7	2132	2371	90	101	19	237	189	119	50	23.2	10	145	27	29.0	116	100	40
8	1608	2150	75	78.9	20	197	156	64.4	36	23.4	13	165	41	27.2	109	500	200
9	1670	2197	76	96.6	23	255	202	59.1	32	11.1	6	209	50	19.2	77	120	48
10	2022	1853	109	88.1	17	236	188	69.9	31	25.3	11	249	49	30.8	123	300	120
11	2236	2850	78	65.7	12	1464	1164	10	41	38.8	16	252	45	30.6	122	0	0
12	1382	2036	68	92.4	27	253	201	55.3	36	18.2	12	160	46	37.1	148	800	320
13	1328	2137	62	66.5	20	201	159	48.7	33	16.3	11	152	46	9.2	37	500	200
14	1780	1623	110	94.4	21	266	212	65.8	33	6.9	3	200	45	17.6	70	220	88
15	1553	2086	74	58.2	15	152	121	45.1	26	14.3	8	234	60	19.3	77	180	72
16	2000	2232	90	105	21	272	217	63.8	29	17.9	8	174	35	20.0	80	60	24
17	1613	1816	89	81.1	20	208	166	53.3	30	22.6	13	210	52	23.3	93	180	72
18	1667	2186	76	75.1	18	180	143	52.5	28	20.4	11	191	46	15.0	60	185	74
19	1825	1947	94	74.5	16	192	153	65.0	32	15.5	8	199	44	12.5	50	0	0
20	1843	1960	94	87.1	19	228	181	71.7	35	19.8	10	191	42	22.1	88	0	0
21	1881	1904	99	107	23	278	221	65.3	31	26.2	13	233	50	29.6	118	0	0
22	2120	2194	97	99.4	19	254	202	59.9	25	21.9	9	289	55	24.0	96	0	0
23	1395	2212	63	67.4	19	174	138	44.4	29	12.3	8	168	48	26.0	104	0	0
24	1772	2026	87	92.7	21	227	181	80.0	41	20.6	10	186	42	27.1	108	250	100
25	1363	1856	73	50.1	15	136	108	42.3	28	11.2	7	158	46	25.4	102	90	36

*Population reference intakes (PRIs), the average requirements (ARs), and adequate intakes (AIs) were taken from Dietary Reference Values for nutrients Summary report by European Food Safety Authority (EFSA) [13]; EER - Estimated Energy Requirement; SFA - saturated fatty acids, EPA - eicosapentaenoic acid, DHA - docosahexaenoic acid

Table S3. Part I. Dietary intake of selected vitamins in subjects with PCOS*

	Vitamin B_1			Vitamin B_2			Vitamin B_3			Vitamin B_6			Folic acid		
No.	Average intake (mg)	AR coverage (%)	PRI coverage (%)	Average intake (mg)	AR coverage (%)	PRI coverage (%)	Average intake (mg)	AR coverage (%)	PRI coverage (%)	Average intake (mg)	AR coverage (%)	PRI coverage (%)	Average intake (μg)	AR coverage (%)	PRI coverage (%)
1	1.1	231	166	1.3	100	81	12.2	142	115	1.2	92	75	271	109	82
2	1.2	172	124	2.2	169	138	19.5	155	126	2.9	223	181	430	172	130
3	1.9	297	214	1.9	146	119	29.3	254	206	2.3	177	144	461	185	140
4	1.2	205	147	1.7	131	106	11.8	112	91	1.6	123	100	377	151	114
5	1.0	255	183	1.0	77	63	17.6	248	202	1.7	131	106	225	90	68
6	0.8	152	109	1.4	108	88	23.5	247	201	2.2	169	138	256	102	78
7	1.2	208	150	1.6	123	100	21.9	211	171	2.0	154	125	369	148	112
8	1.4	350	252	1.3	100	81	26.5	367	298	2.7	208	169	327	131	99
9	1.1	232	167	1.2	92	75	8.4	98	80	1.4	108	88	127	51	39
10	1.3	160	115	3.2	246	200	37.5	256	208	2.4	185	150	475	190	144
11	1.1	193	139	2.3	177	144	25.4	246	200	2.1	162	131	427	171	130
12	1.1	182	131	1.2	92	75	25.4	233	189	2.3	177	144	275	110	84
13	0.8	175	126	0.9	69	56	9.7	118	96	0.9	69	56	153	62	47
14	1.5	217	156	1.4	108	88	26.7	214	174	2.2	169	138	375	150	114
15	2.0	291	210	2.2	169	138	30.4	245	199	3.6	277	225	491	197	149
16	1.2	153	110	2.5	192	156	20.4	144	117	2.7	208	169	480	192	146
17	0.8	148	106	1.6	123	100	14.2	145	118	1.8	138	113	365	146	111
18	0.6	186	134	1.0	77	63	10.7	184	150	1.5	115	94	267	107	81
19	0.9	153	110	1.8	138	113	24.7	232	189	2.5	192	156	436	175	132
20	2.0	376	271	2.1	162	131	34.1	355	289	3.5	269	219	412	165	125
21	1.3	224	161	2.0	154	125	17.0	162	132	1.7	131	106	711	285	216
22	1.3	245	176	1.7	131	106	10.2	106	87	1.5	115	94	265	106	80
23	1.2	215	155	1.0	77	63	10.4	103	84	1.1	85	69	225	90	68
24	1.7	219	157	1.4	108	88	17.0	121	98	2.5	192	156	454	182	138
25	1.6	276	199	1.5	115	94	25.9	248	201	2.1	162	131	294	118	89

*Population reference intakes (PRIs), the average requirements (ARs), and adequate intakes (AIs) were taken from Dietary Reference Values for nutrients Summary report by European Food Safety Authority (EFSA) [13]

Table S3. Part II. Dietary intake of selected vitamins in subjects with PCOS*

No.	Vitamin B_{12} Average intake (μg)	Vitamin B_{12} AI coverage (%)	Vitamin C Average intake (mg)	Vitamin C AR coverage (%)	Vitamin C PRI coverage (%)	Vitamin A (retinol equivalent) Average intake (μg)	Vitamin A (retinol equivalent) AR coverage (%)	Vitamin A (retinol equivalent) PRI coverage (%)	Vitamin D Average intake (μg)	Vitamin D AI coverage (%)	Vitamin E Average intake (μg)	Vitamin E AI coverage (%)
1	4.4	110	44.6	56	47	567	116	87	7.4	49	8.2	75
2	4.4	110	214	268	226	1836	375	282	7.5	50	13.1	119
3	4.1	103	246	309	260	1938	396	298	1.9	13	13.4	122
4	3.2	80	151	190	160	583.1	119	90	2.8	19	9.6	87
5	3.3	83	93.3	117	98	1020	208	157	2.7	18	9.2	84
6	3.4	85	158	198	167	1067	218	164	1.1	7	9.5	86
7	3.4	85	75.4	94	79	1215	248	187	2.6	17	13.5	123
8	4.3	108	184	230	194	1411	288	217	6.1	41	10.2	93
9	1.3	33	132	166	139	461	94	71	1.1	7	6.6	60
10	4.2	105	149	187	157	1411	288	217	4.7	31	14.4	131
11	2.6	65	121	152	128	888	181	137	2.1	14	11.4	104
12	1.4	35	102	128	108	1079	220	166	1.4	9	7.5	68
13	1.5	38	24.3	30	26	402	82	62	1.9	13	3.9	35
14	1.3	33	115	145	122	539	110	83	1.4	9	9.4	85
15	3.8	95	241	302	254	1123	229	173	2.8	19	14.6	133
16	3.6	90	168	211	177	903	184	139	2.7	18	14.4	131
17	3.5	88	157	196	165	1023	209	157	3.2	21	9.4	85
18	1.9	48	150	188	158	1639	335	252	2.5	17	5.9	54
19	3.5	88	271	339	285	1434	293	221	3.2	21	14.6	133
20	3.5	88	169	212	178	1318	269	203	3.3	22	10.2	93
21	2.3	58	222	278	234	1386	283	213	2.7	18	17.4	158
22	2.7	68	118	148	125	297	61	46	4	27	5.7	52
23	2.3	58	152	190	160	600	122	92	2.2	15	8.1	74
24	2.6	65	243	305	256	1165	238	179	5	33	16.5	150
25	2.4	60	127	160	134	603	123	93	2.1	14	11.7	106

*Population reference intakes (PRIs), the average requirements (ARs), and adequate intakes (AIs) were taken from Dietary Reference Values for nutrients Summary report by European Food Safety Authority (EFSA) [13]

Table S4. Part I. Dietary intake of selected vitamins in subjects without PCOS*

	Vitamin B_1			Vitamin B_2			Vitamin B_3			Vitamin B_6			Folic acid		
No.	Average intake (mg)	AR coverage (%)	PRI coverage (%)	Average intake (mg)	AR coverage (%)	PRI coverage (%)	Average intake (mg)	AR coverage (%)	PRI coverage (%)	Average intake (mg)	AR coverage (%)	PRI coverage (%)	Average intake (µg)	AR coverage (%)	PRI coverage (%)
1	1.8	334	240	1.7	131	106	11.5	118	96	1.9	146	119	381	152	115
2	1.3	235	169	1.5	115	94	19.7	197	160	2.1	162	131	326	131	99
3	1.3	214	154	1.7	131	106	14.7	134	109	2.1	162	131	509	204	155
4	0.9	192	138	1.0	77	63	14.5	171	139	1.7	131	106	304	122	92
5	1.5	306	220	1.1	85	69	15.5	175	142	2	154	125	252	101	77
6	1.0	219	157	1.3	100	81	14.5	176	143	1.6	123	100	326	131	99
7	0.5	78	56	1.0	77	63	13.6	117	95	1.2	92	75	286	114	87
8	1.3	268	193	1.3	100	81	17.0	194	158	1.8	138	113	257	103	78
9	0.7	143	103	1.3	100	81	21.3	234	191	1.6	123	100	397	159	120
10	1.4	230	165	1.5	115	94	21.9	199	162	2.2	169	138	356	142	108
11	1.0	148	107	1.5	115	94	12.7	104	85	1.3	100	81	292	117	89
12	1.0	240	173	2.1	162	131	20.3	270	219	2.3	177	144	234	94	71
13	0.7	175	126	1.0	77	63	14.9	206	168	1.2	92	75	163	65	50
14	1.0	186	134	1.1	85	69	14.7	152	123	1.3	100	81	216	87	66
15	1.0	214	154	1.2	92	75	12.3	146	118	1.1	85	69	342	137	104
16	0.8	133	96	1.7	131	106	14.7	135	110	1.9	146	119	252	101	76
17	1.3	268	193	2.1	162	131	21.7	247	201	2.2	169	138	371	149	113
18	1.1	219	158	1.4	108	88	16.6	183	149	1.6	123	100	299	120	91
19	0.5	91	65	1.0	77	63	9.9	100	81	1.2	92	75	248	100	75
20	1.1	198	143	1.1	85	69	22.3	222	181	1.9	146	119	348	139	106
21	1.0	176	127	2.1	162	131	20.9	204	166	1.3	100	81	522	209	158
22	1.2	188	135	1.7	131	106	15.7	136	111	1.7	131	106	284	114	86
23	1.1	262	188	1.0	77	63	21.3	281	228	2.3	177	144	323	129	98
24	1.0	187	135	1.5	115	94	19.1	198	161	2.1	162	131	314	126	95
25	1.0	244	175	1.0	77	63	22.2	299	243	2.4	185	150	421	169	128

*Population reference intakes (PRIs), the average requirements (ARs), and adequate intakes (AIs) were taken from Dietary Reference Values for nutrients Summary report by European Food Safety Authority (EFSA) [13]

Table S4. Part II. Dietary intake of selected vitamins in subjects without PCOS*.

	Vitamin B_{12}		Vitamin C			Vitamin A (retinol equivalent)			Vitamin D		Vitamin E	
No.	Average intake (µg)	AI coverage (%)	Average intake (mg)	AR coverage (%)	PRI coverage (%)	Average intake (µg)	AR coverage (%)	PRI coverage (%)	Average intake (µg)	AI coverage (%)	Average intake (µg)	AI coverage (%)
1	2.4	60	201	251	212	1512	309	233	3.4	23	11.7	106
2	3.3	83	92.3	115	97	783	160	121	2.8	19	9.7	88
3	3.2	80	204	255	215	1223	250	188	5.1	34	4.5	41
4	1.5	38	144	181	153	1418	289	218	1.3	9	8.8	80
5	3.6	90	133	166	140	713	146	110	7.4	49	6.6	60
6	1.8	45	119	149	125	1145	234	176	0.8	5	8.2	75
7	5.2	130	184	230	194	1248	255	192	5.6	37	9.2	84
8	4.7	118	62.5	78	66	1134	231	174	2.3	15	8.0	73
9	3.2	80	90.1	113	95	839	171	129	2.3	16	8.6	78
10	2.7	68	139	175	147	1173	239	180	2.9	19	9.8	89
11	1.8	45	128	160	135	1007	206	155	2.3	15	10.8	98
12	5.2	130	233	292	245	977	199	150	1.3	9	13.4	122
13	2.6	65	39.0	49	41	620	127	95	4.5	30	5.6	51
14	2.7	68	95.8	120	101	1203	246	185	2.6	17	11.1	101
15	3.4	85	78.4	98	83	393	80	61	1.2	8	8.3	75
16	5.7	143	89.3	112	94	660	135	102	6.2	41	6.5	59
17	2.1	53	137	172	145	992	202	153	2.4	16	7.8	71
18	5.6	140	71.3	89	75	1913	390	294	1.0	7	6.3	57
19	2.0	50	58.8	74	62	393	80	61	1.4	9	4.5	41
20	2.3	58	80.1	100	84	1189	243	183	1.6	11	8.3	75
21	6.6	165	228	286	241	1323	270	204	3.2	21	12.6	115
22	2.9	73	87.4	109	92	724	148	111	1.6	11	8.0	73
23	2.3	58	160	201	169	1290	263	198	1.2	8	10.3	94
24	3.0	75	125	157	133	1351	276	208	3.1	21	8.5	77
25	2.6	65	172	216	182	1281	261	197	2.8	19	13.4	122

*Population reference intakes (PRIs), the average requirements (ARs), and adequate intakes (AIs) were taken from Dietary Reference Values for nutrients Summary report by European Food Safety Authority (EFSA) [13]

Table S5. Part I. Dietary intake and AR, PRI and AI coverage (%) of selected minerals in subjects with PCOS*

	Sodium#		Potassium		Calcium			Phosphorus		Magnesium	
**No.**	**Average intake (mg)**	**AI# (%)**	**Average intake (mg)**	**AI (%)**	**Average intake (mg)**	**AR (%)**	**PRI (%)**	**Average intake (mg)**	**AI (%)**	**Average intake (mg)**	**AI (%)**
1	1801	90	1884	54	751	100	79	1164	212	275	92
2	1999	100	3694	106	730	85	73	1677	305	412	137
3	2638	132	4187	120	763	102	80	1537	279	364	122
4	1666	83	2919	83	981	114	98	1403	255	349	117
5	2364	118	2666	76	532	62	53	1029	187	261	87
6	2377	119	2351	67	602	70	60	1246	227	462	154
7	1686	84	2901	83	603	80	64	1257	229	272	91
8	1512	76	3182	91	413	55	43	1164	212	283	95
9	1246	62	2060	59	249	33	26	478	87	161	54
10	2466	123	3641	104	936	125	99	1996	363	460	153
11	2328	116	3492	100	599	80	63	1378	251	370	123
12	2409	120	2943	84	1417	165	142	1556	283	343	114
13	2777	139	1315	38	368	49	39	997.6	181	188	63
14	1944	97	4099	117	585	68	59	1506	274	450	150
15	2494	125	4456	127	939	125	99	1778	323	491	164
16	2169	108	4107	117	1019	118	102	1762	320	461	154
17	1551	78	2582	74	648	75	65	1222	222	258	86
18	1950	98	2029	58	338	39	34	739	134	157	52
19	2388	119	3607	103	628	73	63	1268	231	322	108
20	1873	94	3519	101	763	102	80	1507	274	389	130
21	1809	90	3776	108	869	116	92	1712	311	483	161
22	1505	75	2185	62	733	98	77	950	173	231	77
23	1328	66	2073	59	390	45	39	744	135	188	63
24	2314	116	4826	138	815	109	86	1267	230	533	178
25	1357	68	3430	98	476	55	48	1299	236	365	122

*Population reference intakes (PRIs), the average requirements (ARs), and adequate intakes (AIs) were taken from Dietary Reference Values for nutrients Summary report by European Food Safety Authority (EFSA) [13], #Dietary reference values for sodium [50]

Table S5. Part II. Dietary intake and AR, PRI and AI coverage (%) of selected minerals in subjects with PCOS*

	Iron			Zinc			Copper		Iodine		Manganese	
No.	Average intake (mg)	AR (%)	PRI (%)	Average intake (mg)	AR (%)	PRI (%)	Average intake (mg)	AI (%)	Average intake (μg)	AI (%)	Average intake (mg)	AI (%)
1	11.5	164	72	9.4	152	125	1.4	108	93.1	62	4.8	160
2	17.2	246	108	12.4	200	165	1.5	115	54.3	36	5.9	197
3	16.0	229	100	10.2	165	136	1.7	131	37.7	25	4.2	140
4	11.9	170	74	8.4	135	112	1.2	92	36.6	24	4.7	157
5	9.1	130	57	8.1	131	108	0.6	46	33.3	22	2.4	80
6	10.3	147	64	8.8	142	117	0.8	62	31.4	21	4.1	137
7	12.9	184	81	9.6	155	128	1.0	77	39.0	26	3.3	110
8	11.5	164	72	7.9	127	105	0.9	69	51.3	34	3.5	117
9	8.4	120	53	4.3	69	57	1.4	108	9.1	6	2.3	77
10	16.6	237	104	12.4	200	165	1.1	85	58.4	39	2.3	77
11	15.2	217	95	11.4	184	152	1.4	108	66.4	44	2.5	83
12	11.4	163	71	10.2	165	136	2.4	185	18.5	12	2.7	90
13	6.2	89	39	6.9	111	92	1.2	92	13.5	9	1.4	47
14	15.3	219	96	11.1	179	148	2.2	169	22.1	15	3.6	120
15	17.5	250	109	12.4	200	165	1.8	138	37.5	25	4.8	160
16	17.4	249	109	13.4	216	179	2.6	200	36.4	24	5.2	173
17	10.3	147	64	8.5	137	113	0.9	69	41.9	28	3.9	130
18	9.4	134	59	6.1	98	81	0.6	46	60.5	40	1.2	40
19	15.1	216	94	9.8	158	131	1.1	85	37.4	25	3.7	123
20	14.6	209	91	11.1	179	148	1.4	108	29.4	20	4.1	137
21	19.7	281	123	13.3	215	177	2.0	154	37.4	25	3.2	107
22	8.6	123	54	7.4	119	99	0.9	69	21.2	14	2.8	93
23	8.9	127	56	6.8	110	91	0.7	54	55.9	37	2.8	93
24	18.6	266	116	9.9	160	132	2.4	185	50.7	34	2.4	80
25	13.4	191	84	8.8	142	117	1.1	85	67.4	45	4.3	143

*Population reference intakes (PRIs), the average requirements (ARs), and adequate intakes (AIs) were taken from Dietary Reference Values for nutrients Summary report by European Food Safety Authority (EFSA) [13]

Table S6. Part I. Dietary intake and AR, PRI and AI coverage (%) of selected minerals in subjects without PCOS*

	Sodium[#]		Potassium		Calcium			Phosphorus		Magnesium	
No.	Average intake (mg)	AI# (%)	Average intake (mg)	AI (%)	Average intake (mg)	AR (%)	PRI (%)	Average intake (mg)	AI (%)	Average intake (mg)	AI (%)
1	3044	152	3042	87	846	98	85	1263	230	306	87
2	1823	91	2785	80	980	114	98	1482	269	359	103
3	1955	98	3305	94	977	114	98	1397	254	457	131
4	1812	91	2286	65	389	45	39	941	171	254	73
5	1070	54	2525	72	486	57	49	1017	185	266	76
6	1555	78	2611	75	699	81	70	1152	209	284	81
7	1391	70	2449	70	464	54	46	956	174	295	84
8	2851	143	2529	72	733	85	73	1394	253	328	94
9	2224	111	2353	67	435	58	46	1181	215	250	71
10	2240	112	3300	94	589	79	62	1357	247	369	106
11	2039	102	2438	70	744	99	78	1345	245	371	106
12	2409	120	3926	112	1030	137	108	1510	275	318	91
13	1951	98	1602	46	421	49	42	824	150	157	45
14	1477	74	2075	59	628	73	63	979	178	228	65
15	1028	51	2505	72	791	92	79	1096	199	261	75
16	1356	68	2681	77	625	73	63	1323	241	242	69
17	1990	100	2929	84	830	111	87	1261	229	1025	293
18	1996	100	2400	69	454	53	45	933	170	234	67
19	1203	60	1736	50	538	63	54	881	160	241	69
20	2294	115	2854	82	604	81	64	1305	237	335	96
21	2117	106	4273	122	737	98	78	1675	305	485	139
22	2396	120	2293	66	762	89	76	1197	218	230	66
23	1340	67	2821	81	642	86	68	1126	205	330	94
24	1869	93	3088	88	534	62	53	1338	243	352	101
25	1124	56	2925	84	563	65	65	1043	190	302	86

* Population reference intakes (PRIs), the average requirements (ARs), and adequate intakes (AIs) were taken from Dietary Reference Values for nutrients Summary report by European Food Safety Authority (EFSA) [13], #Dietary reference values for sodium [50]

Table S6. Part II. Dietary intake and AR, PRI and AI coverage (%) of selected minerals in subjects without PCOS*

	Iron			Zinc			Copper		Iodine		Manganese	
**No.**	**Average intake (mg)**	**AR (%)**	**PRI (%)**	**Average intake (mg)**	**AR (%)**	**PRI (%)**	**Average intake (mg)**	**AI (%)**	**Average intake (µg)**	**AI (%)**	**Average intake (mg)**	**AI (%)**
1	13.9	199	87	9.6	155	128	1.2	92	44.6	30	4.0	133
2	13.5	193	84	10.6	171	141	1.4	108	35.4	24	5.5	183
3	12.2	174	76	9.6	155	128	1.6	123	65.7	44	7.1	237
4	9.9	141	62	7.2	116	96	1.0	77	24.8	17	4.1	137
5	10.8	154	68	8.0	129	107	1.1	85	15.6	10	4.5	150
6	10.5	150	66	7.9	127	105	1.0	77	29.2	19	4.7	157
7	11.2	160	70	8.2	132	109	1.1	85	44.5	30	5.7	190
8	12.3	176	77	11.2	181	149	1.1	85	33.3	22	6.6	220
9	10.0	143	62	9.0	145	120	1.0	74	51.5	34	3.2	108
10	13.3	190	83	10.7	173	143	1.3	100	36.2	24	6.3	210
11	12.0	171	75	10.7	173	143	1.3	100	19.7	13	6.2	207
12	13.7	196	86	15.2	245	203	1.3	100	53.6	36	4.2	140
13	5.4	77	34	4.6	74	61	0.5	38	18.1	12	1.0	33
14	9.3	133	58	7.3	118	97	0.6	46	24.8	17	2.6	87
15	8.9	127	56	7.2	116	96	1.2	92	34.4	23	2.5	83
16	9.8	140	61	9.3	150	124	1.0	77	41.9	28	2.4	80
17	12.1	173	76	8.3	134	111	1.1	85	31.4	21	2.5	83
18	10.0	143	63	7.2	116	96	0.9	69	20.9	14	2.3	77
19	8.4	120	53	6.3	102	84	1.1	85	18.1	12	5.0	167
20	12.7	181	79	9.1	147	121	1.0	77	39.7	26	3.5	117
21	16.7	239	104	13.2	213	176	2.2	169	58.3	39	4.5	150
22	9.4	134	59	8.1	131	108	0.9	69	21.4	14	2.4	80
23	11.1	159	69	8.7	140	116	1.2	92	34.9	23	1.8	60
24	10.7	153	67	9.0	145	120	1.1	85	22.5	15	3.3	110
25	11.4	163	71	8.8	142	117	1.2	92	25.6	17	2.7	90

*Population reference intakes (PRIs), the average requirements (ARs), and adequate intakes (AIs) were taken from Dietary Reference Values for nutrients Summary report by European Food Safety Authority (EFSA) [13]
